# Why Internists Should Care About Dry Eye Disease

**DOI:** 10.3390/jcm9020532

**Published:** 2020-02-15

**Authors:** Anat Galor

**Affiliations:** Ophthalmology, Miami Veterans Affairs Medical Center, Bascom Palmer Eye Institute, University of Miami, Miami, FL 33125, USA; agalor@med.miami.edu

Dry eye disease (DED) has been diagnosed and managed under the purview of the eye care professional, with internists typically not paying much attention to the condition. This Journal of Clinical Medicine’s Special Issue entitled "Dry Eye Syndrome: New Insights on Epidemiology and Management" highlights why internists should care about DED. First, DED is a common condition, with DE symptoms being found in 5%–30% of the population, increasing in frequency with increasing age [1,2]. This means that many patients seen by internists have DE symptoms, including both painful symptoms (e.g., sensations of dryness, burning, aching, foreign body sensation) and visual complaints (e.g., fluctuating or poor vision). Second, DE symptoms tend to be chronic and negatively impact quality of life (QoL), with impairments in reading, driving, and computer use described [1,3]. As such, internists can work with eye care providers to identify and manage individuals with DED, leading to improvements in QoL. 

The internist needs to understand several aspects of DED when considering the impact of the disease on their patients. First, it is important to realize that DED is not one disease but instead, a “multi-factorial disease of the ocular surface characterized by a loss of homeostasis of the tear film, and accompanied by ocular symptoms, in which tear film instability and hyperosmolarity, ocular surface in-flammation and damage, and neurosensory abnormalities play etiological roles”[4]. In fact, in contrast to what is suggested by the terminology, most individuals with DED do not have a deficit in tear production, although this sub-type of DED (i.e., aqueous tear deficiency) is commonly encountered with individuals with Sjögren’s and Graft versus Host Disease [1]. To add to this complexity, sensations of dryness and other DE symptoms (e.g., burning, aching) are often disconnected from tear and ocular surface findings (e.g., decreased tear production, increased tear evaporation, corneal epithelial disruption) [5]. In fact, certain systemic disorders, such as fibromyalgia, migraine, and traumatic brain injury, are oftentimes seen in individuals whose DE symptoms outweigh ocular surface signs [6]. On the other hand, individuals with diabetes mellitus, for example, often present with a plethora of DE signs (e.g., corneal epithelial disruption) with minimal DE symptoms. As such, the sub-type of DED is often aligned with its accompanying systemic co-morbidities. For example, as noted above, aqueous tear deficiency is often seen in Sjögren’s disease, while evaporative dry eye often accompanies rosacea [7]. Furthermore, these associations often guide DED treatment [8]. For example, blood-based products (e.g., serum tears) are more frequently used in individuals with a systemic immune disorder [9]. As such, treatments of DE must consider both ocular and systemic findings when formulating therapeutic algorithms. 

This Special Issue highlights several inter-connections between DED and systemic health and highlights why internists are an important part of the DED conversation. 

First, it is important to understand that DED (symptoms and/or signs) can occur both as an isolated disease of the ocular surface or as part of a systemic disorder. The former is often seen in individuals with glaucoma who use topical anti-hypertensives [10] and in individuals whose DED commenced after eye surgery [11,12]. The latter has been described in conditions such as Sjögren’s and other auto-immune diseases, thyroid disease, peptic ulcer disease, sleep apnea [1], and, highlighted in this issue, the association between DED and gout [13]. Using the National Health Insurance Research Database (NHIRD), researchers have found that a diagnosis of gout portended a 6.5% increased risk of DED development (via International Classification of Diseases, Ninth Revision) after adjusting for potential co-morbidities, and this risk positively correlated with a longer disease period [13]. Internists can use this information to identify individuals at higher risk for DED and refer those with persistent symptoms to an eye care provider. This is important as chronic DE symptoms are a source of significant morbidity, negatively impacting physical and mental function [3]. A Japanese study investigated the characteristics of individuals with undiagnosed DED, defined as frequent DED symptoms without a clinical diagnosis. A multivariate adjusted model found that younger age, female sex, and prolonged visual display terminal usage were risk factors for undiagnosed DED. Furthermore, individuals with undiagnosed DED who tried less than three over-the-counter therapies had significantly worse DE-related QoL compared to individuals with diagnosed DED [14]. These data suggest that the appropriate referral of symptomatic patients to an eye care provider can improve dry eye associated QoL.

Second, DE symptoms may be a local manifestation of a systemic disorder and eye care providers often need the help of internists to manage systemic manifestations of disease. For example, many individuals with chronic DE symptoms have co-morbid diabetes, fibromyalgia or migraines that need to be managed [6]. Furthermore, individuals with chronic DE symptoms often have co-morbid depression and anxiety. For example, a study in the Special Issue noted that individuals with DE symptoms had dysfunctional coping mechanisms [15]. Specifically, catastrophizing scores via the Pain Catastrophizing Scale (PCS) were correlated with DE symptom severity, including the Dry-Eye Questionnaire 5 (*r* = 0.41, *p* < 0.0005), Ocular Surface Disease Index (*r* = 0.40, *p* < 0.0005), and neuropathic-like eye pain (Neuropathic Pain Symptom Inventory-Eye [16]; *r* = 0.48, *p* < 0.0005). The magnitude of the findings was in the order of what has been described in chronic pain conditions outside the eye. These findings highlight that, as with chronic non-ocular pain, comprehensive and multidisciplinary patient-centered approaches delivered by a team of expert clinicians are necessary to achieve optimal outcomes in patients with DED. 

Third, eye care providers can use information generated by internists to formulate better preventative and therapeutic algorithms for patients. Preemptive analgesia is an approach that has been used in multiple surgeries that protects nerves at the time of surgical damage, with immediate and late effects on pain. As detailed in the Special Issue, one group applied this concept to refractive eye surgery. Specifically, in a randomized placebo-controlled study, oral pregabalin was administered, starting with one dose prior to laser-assisted in situ keratomileusis (LASIK) and continued for 28 doses (14 days) with the outcome being the effect of the intervention on frequency and severity of DE symptoms 6 months after surgery. While the study did not find a benefit to perioperative oral pregabalin [11], it demonstrated that ideas successful in preventing and treating non-ocular conditions could be modified and tested for the prevention and treatment of eye diseases.

Fourth, tears can be used as biomarkers of local and systemic disease. In this special issue, one group demonstrated that tear IgE levels could be measured in individuals with DE symptoms but without known allergic conjunctivitis [17]. Several risk factors for allergic conjunctivitis (pets in the home, smoking) correlated with elevated tear IgE levels, suggesting that some individuals labeled as having DED may have an underlying allergic component. This line of research opens up the possibility of using tears as a diagnostic fluid for other systemic diseases.

To conclude, DED is a common ocular disease with significant morbidity. It can be an isolated disease of the ocular surface or can occur co-morbid with systemic findings. The internist and eye care provider can form an important partnership to identify and treat DED, using a holistic approach that takes into consideration both ocular and systemic co-morbidities.
